# Effect of *Carica papaya* on IRS-1/Akt Signaling Mechanisms in High-Fat-Diet–Streptozotocin-Induced Type 2 Diabetic Experimental Rats: A Mechanistic Approach

**DOI:** 10.3390/nu14194181

**Published:** 2022-10-08

**Authors:** Jeane Rebecca Roy, Coimbatore Sadagopan Janaki, Selvaraj Jayaraman, Vijayalakshmi Periyasamy, Thotakura Balaji, Madhavan Vijayamalathi, Vishnu Priya Veeraraghavan

**Affiliations:** 1 Department of Anatomy, Bhaarath Medical College and Hospital, Bharath Institute of Higher Education and Research (BIHER), Chennai 600 073, Tamil Nadu, India; 2 Centre of Molecular Medicine and Diagnostics (COMManD), Department of Biochemistry, Saveetha Dental College & Hospital, Saveetha Institute of Medical & Technical Sciences, Chennai 600 077, Tamil Nadu, India; 3 Department of Biotechnology and Bioinformatics, Holy Cross College, Trichy 620 002, Tamil Nadu, India; 4 Department of Anatomy, Chettinad Hospital and Research Institute, Chettinad Academy of Research and Education, Chennai 603 103, Tamil Nadu, India; 5 Department of Physiology, Bhaarath Medical College and Hospital, Bharath Institute of Higher Education and Research (BIHER), Chennai 600 073, Tamil Nadu, India

**Keywords:** *Carica papaya*, insulin receptor substrate-1, Akt, skeletal muscle, insulin signaling, molecular dynamics

## Abstract

Despite rigorous endeavors, existing attempts to handle type 2 diabetes (T2DM) are still a long way off, as a substantial number of patients do not meet therapeutic targets. Insulin resistance in skeletal muscle is discerned as a forerunner in the pathogenesis of T2DM and can be detected years before its progress. Studies have revealed the antidiabetic properties of *Carica papaya* (*C. papaya*), but its molecular mechanism on insulin receptor substrate-1 (IRS-1)/Akt signaling mechanisms is not yet known. The present study aimed to evaluate the role of *C. papaya* on IRS1 and Akt in high-fat-diet–streptozotocin-induced type 2 diabetic rats and also to analyze the bioactive compounds of *C. papaya* against IRS-1 and Akt via in silico analysis. Ethanolic extract of the leaves of *C. papaya* (600 mg/kg of body weight) was given daily for 45 days postinduction of T2DM up to the end of the study. Gluconeogenic enzymes, glycolytic enzymes, gene expression, and immunohistochemical analysis of IRS-1 and Akt in skeletal muscle were evaluated. *C. papaya* treatment regulated the levels of gluconeogenic and glycolytic enzymes and the levels of IRS-1 and Akt in skeletal muscle of type 2 diabetic animals. In silico studies showed that trans-ferulic acid had the greatest hit rate against the protein targets IRS-1 and Akt. *C. papaya* restored the normoglycemic effect in diabetic skeletal muscle by accelerating the expression of IRS-1 and Akt.

## 1. Introduction

The incidence of type 2 diabetes mellitus (T2DM) has stretched to epidemic levels globally, which has led to a substantial impact on human life and health economics. Despite significant efforts, currently accessible treatments and preventive measures for T2DM are far from ideal, and a significant number of patients do not achieve treatment objectives [1]. Hence, much research and efforts still lie ahead to comprehend the pathogenesis of T2DM and reduce the advancement of prediabetes to diabetes [2]. A rising demand for handling T2DM has been demonstrated in recent years, and this demand still persists.

Insulin resistance in skeletal muscle is an early impairment in the pathogenesis of T2DM and can be identified years before the onset of this metabolic syndrome [3]. Almost 80% of circulating glucose after a postprandial meal is taken up by the cardinal site, skeletal muscle. The glucose passes from the extracellular matrix to the cell membrane, and subsequently, with the aid of glucose transporters, it is ingested into the cell. Intracellular metabolism regulates the glucose gradient to improve the transport of glucose [4]. Impairment in glucose uptake and disposal in skeletal muscle ultimately lead to defects in whole-body glucose uptake due to diminished insulin-stimulated glycogen synthesis led by flaws in glycogen synthase and glucose transport [5]. An inactive way of life and hyperalimentation mark a rise in free fatty acids and inflammatory cytokines that cause inflammation and oxidative impairment, diminishing the potential of skeletal muscle in response to insulin needs [4,6]. Endothelial malfunction and deposition of matrix proteins occur in skeletal muscle due to hyperalimentation, subsequently modifying insulin signaling and thus altering normal metabolism in glucose absorption [4,7]. Free radicals of reactive oxygen and nitrogen species reduce antioxidant levels and alter other biomolecules, eventually oxidizing proteins, lipids, and nucleic acids. Toxic derivatives are set off, eliciting endothelial cell damage and tissue dysfunction in type 2 diabetes, reducing the cell’s potential to react to stimuli [8,9,10].

Once insulin binds with its receptor, tyrosine residues are autophosphorylated, causing tyrosine phosphorylation of IRS-1 and IRS-2, which leads to associating and triggering the phosphatidylinositol-3 kinase (PI3K) enzyme [11]. PIP3 increases as a result of subsequent phosphorylation and activation of the p110 subunit, which in turn regulates the activity of phosphoinositide-dependent kinase-1 (PDK-1). Protein kinase B/Akt is triggered to phosphorylate AS160, which enables GLUT4 to bind to the sarcolemma by either diminishing the tethering of the GLUT4 vesicle by TUG proteins or by enhancing Rab proteins to induce GLUT4 vesicle translocation to the sarcolemma [6,12]. The veracity of the IRS-1/PI-3 kinase/Akt pathway is effectively upheld for normal insulin-affected glucose uptake in skeletal muscle [6,13].

Serine/threonine residue phosphorylation occurs instead of tyrosine phosphorylation in the state of insulin resistance and in due course alters downstream effectors such as Akt and atypical PKC, subsequently diminishing the translocation of GLUT4 and reducing glucose uptake [14]. Insulin resistance can occur due to fat accumulation in skeletal muscle due to the imbalance of uptake and oxidation of fatty acids, subsequently causing metabolic inflexibility [15]. Normal individuals bank on fat oxidation under fasting conditions and shift effortlessly to revert carbohydrate oxidation to insulin stimulation, which is mislaid in the insulin resistance state [16].

Hyperglycemic-induced oxidative stress triggers serine-threonine kinases such as IKKβ that sequentially phosphorylate IR and IRS-1 and in succession downregulate PI3K initiation to instigate insulin resistance [17,18]. Insulin-regulated IRS tyrosine phosphorylation is vital in insulin signaling. One of the targets to induce insulin resistance and IRS-1 at a low ebb is viewed in obese and insulin-resistant individuals [19,20]. In skeletal muscle, IRS-1-dependent insulin signal activity prevails over IRS2 to sustain metabolism [21]. IRS-1 is highly mutant, and these alterations reduce IRS-1 phosphorylation and insulin-induced PI3K activity, which induces debilitated insulin action [21,22,23]. There are various factors, such as free fatty acids, inflammatory cytokines, ROS, and hyperinsulinemia, that hike the different serine kinases that can hinder IRS-1 activity. These manifest insulin resistance due to upregulation of gene expression by triggering the inflammation process and nuclear factor kappa B (NF-ĸB) [19,23,24].

Akt plays a major part in insulin-initiated glucose absorption, glycogen synthesis, and even cell growth and survival. Threonine 308 is phosphorylated by phosphoinositide-dependent protein kinase 1 (PDK1) and is followed by phosphorylation at serine 473 by PI3K via mTOR complex 2 to activate Akt for GLUT4 translocation and glucose uptake [25,26]. Previous studies have shown that knockdown of Akt1 or Akt 2 can result in insulin resistance and glucose intolerance. Akt diminishes the transcriptional activity of FOXO1 and reduces glucose levels [27,28].

Therefore, targeting factors and receptors related to skeletal muscle insulin action can help improve insulin resistance in type 2 diabetes to prevent further progression. Medicinal plants are scrutinized meticulously in handling diabetes mellitus in order to evade the after effects of modern medicine. In our previous study, we focused on the effect of *Carica papaya* (*C. papaya*) on insulin signaling targets such as IR and GLUT4 in skeletal muscle of type 2 diabetes, as well as in silico analysis of the bioactive compounds of *C. papaya* against IR and GLUT-4, its enhancement in glucose uptake, and decrease in insulin resistance. The present study concentrated on the effect of *C. papaya* on IRS-1 and Akt in vivo and in silico to understand the role of *C. papaya* in insulin signaling and gene expression analysis in skeletal muscle of high-fat-diet (HFD)–streptozotocin-induced T2DM experimental Wistar rats.

## 2. Materials and Methods

### 2.1. Chemicals and Reagents

All chemicals, reagents, and primers used in this study were procured from Sigma Chemical Company (St. Louis, MO, USA), Crystal Chem Inc. (Elk Grove Village, IL, USA), MP Biomedicals (Santa Ana, CA, USA), Invitrogen (United States), New England Biolabs (NEB) (United States), Promega (United States), and Eurofins Genomics India Pvt Ltd. (Bangalore, India).

### 2.2. Collection of Plant Material

Leaves of *C. papaya* were collected from Kerala. They were shade dried and powdered. The material was authenticated by the National Institute of Siddha, Chennai (Certificate No.: NISMB4392020).

### 2.3. Animals

Adult male Wistar albino rats (150–180 days old) were maintained under standard environmental conditions of standard temperature and specific humidity (21 ± 2 °C), a continual 12 h dark and 12 h light cycle, according to the Institutional Animal Ethical Committee. They were fed a standard pellet diet and water ad libitum at Central Animal House, Saveetha Dental College and Hospital, Chennai, Tamil Nadu. The present work was approved according to current guidelines (IAEC No.: BRULAC/SDCH/SIMATS/IAEC/08-2021/071, dated 21 August 2021).

### 2.4. Induction of T2DM

A high-fat diet (HFD) (66% typical rat feed, 3% cholesterol, 1% cholic acid, and 30% coconut oil) was catered to the rats for 4 weeks. After 4 weeks of high-fat-diet (HFD) feeding, rats were injected intraperitoneally with a low dose of streptozotocin (STZ) 35 mg/kg (Sigma Aldrich, St. Louis, MO, USA) [29]. Following the next two days of STZ injection, rats with a fasting blood glucose level (>120 mg/dL) were considered for the experiment. Diabetic rats were allowed to feed HFD and sucrose water during the study.

### 2.5. Experimental Design

Rats were randomly divided into 5 groups of 8 rats each.

Group 1—Control rats; Group 2—Diabetic rats; Group 3—Diabetic rats +600 mg/kg bwt ethanolic extract of *C. papaya* for 45 days; Group 4—Diabetic rats +50 mg/kg bwt of metformin for 45 days; Group 5—Control +600 mg/kg bwt ethanolic extract of *C. papaya* for 45 days.

On the last day of the experiment, the animals were sedated with sodium thiopentone (40 mg/kg body weight); blood was drawn via cardiac puncture, and sera were separated and stored at −80 °C. Blood was removed from the organs by injecting 20 mL of isotonic sodium chloride solution through the left ventricle. The gastrocnemius muscle was dissected instantly and taken for the following parameters.

### 2.6. Determination of Gluconeogenic Enzymes

#### 2.6.1. Glucose-6-Phosphatase Assay

The method of Koide and Oda was employed to evaluate glucose-6-phosphatase [30]. A 0.3 mL volume of citrate buffer, 0.5 mL of substrate, and 0.1 mL of homogenate tissue were incubated for 1 h at 37 °C. Then, 10% TCA was added to halt the reaction following the approach of Fiske and Subbarow [31]. The value of absorbance was taken at 640 nm.

#### 2.6.2. Fructose-1,6 Bisphosphatase Assay

The method of Gancedo and Gancedo was used [32]. Incubation for the final mixture (2.3 mL) contained Tris-HCl buffer, substrate, magnesium chloride, potassium chloride, EDTA, and tissue homogenate for 15 mins at 37 °C. Then, 10% TCA halted the reaction. Later estimation followed the Fiske and Subbarow method [31].

### 2.7. Determination of Glycolytic Enzymes

Hexokinase (HK) activity was assessed using the method described by Brandstrup et al. [33]. HK produced glucose 6-phosphate and ADP from ATP and D-glucose, respectively. When the o-toluidine reagent was applied, the remaining glucose reacted and produced a green color that could be seen at 640 nm. Enzyme’s activity was calculated as mol glucose phosphorylated/h/mg of protein. The approach of Valentine and Tanaka was used to measure the pyruvate kinase (PK) tissue activity [34]. As an initial point, pyruvate production from phosphoenolpyruvate was used. Dinitrophenyl hydrazine was added, and the color that emerged at 520 nm was evaluated to estimate the amount of pyruvate released. Values were denoted as μmol pyruvate formed/min/mg protein.

### 2.8. mRNA Expression Analysis

#### Total RNA Isolation, cDNA Conversion, and Real-Time PCR

Using a TRIR kit, total RNA was separated into five groups. The reverse transcriptase kit was obtained from Eurogentec (Seraing, Belgium). cDNA was made from 2 µg of total RNA. The sequence of the primers used in this study is given in Table 1. The reference gene used is β-actin. Genes were amplified in a real-time PCR system (Stratagene MX 3000P, Agilent Technologies, 530l, Stevens Creek Blvd, Santa Clara, CA 95051, USA) under the following reaction conditions: initial denaturation at 95 °C for 5 min followed by 40 cycles of 95 °C for 30 s, 59–60 °C for 30 s and 72 °C for 30 s. Relative quantification was calculated from the melt and amplification curves analysis.

### 2.9. Immunohistochemical Analysis

Sections of skeletal muscle of about 4 µm from experimental animals were deparaffinized and rehydrated with xylene and ethanol at progressively lower percentages. Specimens were mixed with sodium citrate buffer (1 M, pH 6.0–6.2) and microwaved for three cycles of 5 min each, separated by 1 min. Slides were then washed for 5 min with 1 M PBS for endogenous blocking. In a dark, humid chamber, peroxidase activity was performed for 10 min with 30% H_2_O_2_, followed by a 5 min wash with 1 M PBS. Slides were cleaned twice with PBS for 5 min each after being blocked with 2% bovine serum albumin (BSA). IRS-1 and Akt primary antibodies were used, and they were diluted 1:100 before being treated with the sections. Specimens were incubated with horseradish peroxidase (HRP) in a humid chamber for 45 min, followed by washing for 5 min with 1 M PBS. By using the 3,3′-diaminobenzidine (DAB) substrate chromogen 3.3 (100 mg, Sigma, United States), 1.2 mL of 30% H_2_O_2_, and 120 mL of PBS for 6 min at 37 °C, the final product was revealed and then rinsed in water for 5 min.

### 2.10. Statistical Analysis

Mean ± standard error mean (SEM) was obtained by the triplicate analysis results of the investigations conducted on control and treatment rats. Experimental data were estimated by means of one-way ANOVA and Duncan’s multiple range test using GraphPad Prism Version 5 to detect substantial variances between mean values. Values with *p* < 0.05 were considered statistically significant.

### 2.11. Molecular Docking

#### 2.11.1. Compound/Ligand Preparation

The structures of the selected phytoconstituents from *C. papaya* (Table 2) were obtained in the structural data format (SDF) from the PubChem database. These SDF files were then produced using the “prepare ligands” module of DSBDS software and filtered using the “Filter by Lipinski Rules” module. This procedure eliminated duplicate entries, computed isomers. and tautomers and created and reduced 3D conformations. BIOVIA is a product of Dassault Systèmes.

#### 2.11.2. Protein Preparation

The PDB was used to download the structures of the human IRS-1 (PDB ID: 1K3A) and Akt (PDB ID: 3QKM) After removing all water molecules, the missing hydrogen atoms were supplied using CHARMm force field’s Prepare protein module.

#### 2.11.3. Molecular Docking Procedure

Molecular docking investigations were conducted using the Discovery Studio module Ligand Fit. The primary stage in docking was protein preparation, and entire ligands were docked into the active area of the receptors. A flood-filling algorithm was used to seek active sites. The active site was demarcated as the portion of the receptor that is within 12 of the ligand’s geometric centroid. A total of 10 poses were formed during docking, and the prime poses were chosen based on the docking score values obtained after energy minimization utilizing smart minimization and a molecule’s optimal orientation in the active site. The formula for calculating the docking score is as follows. A consensus scoring system was developed because a single docking score may not be enough to detect active compounds. LigScore1, LigScore2, Jain, Piecewise Linear Potential (PLP1 and PLP2), and Potential of Mean Force (PMF) were used. The active compounds were chosen using a consensus-scoring algorithm and their H-bond interaction with the receptor.

### 2.12. Molecular Simulation and Dynamics

#### Molecular Simulation and Dynamics Study of Proposed Compounds and IRS-1 and Akt Complex

All atom MD simulations were run for 100 ns on all receptors in their free state (apo), as well as docked complexes at 300 K using the GROMOS 54A7 force field in the GROMACS simulation program [38]. The Discovery Studio platform was used to search for and add missing residues in the receptor coordinate file. The apo and docked complexes were solvated in a cubic box (size 1.0 nm) and neutralized with sodium ions using the SPC water model. The PRODRG server was used to build an MD-based ligand topology file for the docked complex [39]. With 1500 ps, the steepest descent approach was utilized to achieve energy minimization. The system temperature was initially fixed to 0 K and subsequently rose to 300 K over the equilibration phase. After that, an equilibration period of 100 ps with constant volume was achieved under periodic boundary conditions with a stable pressure of 1 bar. Graphs were generated using MD simulation data using Xmgrace [40]. For all systems, the final MD run lasted 100 ns, and the resulting trajectories were assessed using GROMACS’s specialized modules. To analyze the stability of the simulation, the root mean square deviation and root mean square fluctuation values, as well as the solvent accessible surface area (SASA) and radius of gyration (RG), were calculated using GROMACS simulation software.

## 3. Results

### 3.1. Estimation of Gluconeogenic Enzymes and Glycolytic Enzymes

**Figure 1:**
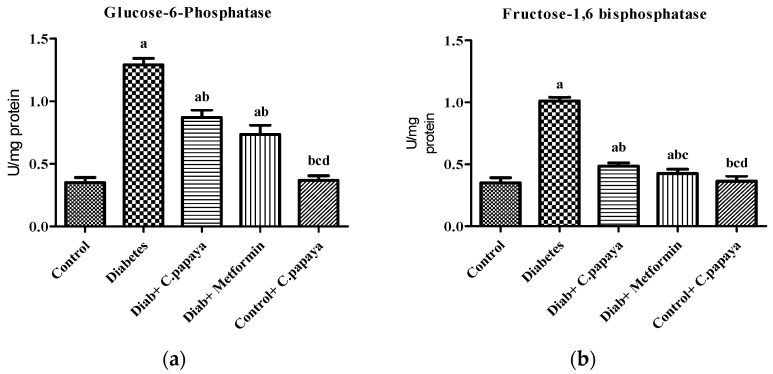
Outcome of ethanolic extract of *C. papaya* on (**a**) glucose-6-phosphatase and (**b**) fructose-1,6 bisphosphatase levels in control and diabetic rats. Each bar illustrates the mean ± SEM of eight rats, with *p* < 0.05 demonstrating significant differences between the groups: a—control; b—diabetes; c—diabetic rats administered with ethanolic extract of *C. papaya;* d—diabetic rats treated with metformin.

**Figure 2:**
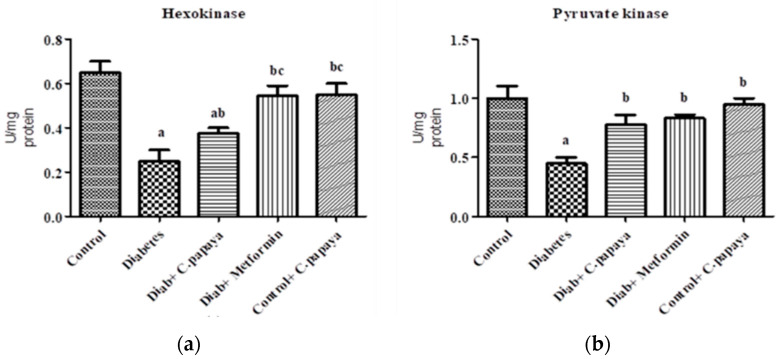
Outcome of ethanolic extract of *C. papaya* on (**a**) hexokinase and (**b**) pyruvate kinase levels in control and diabetic rats. Each bar illustrates the mean ± SEM of eight rats, with *p* < 0.05 demonstrating significant differences between the groups: a—control; b—diabetes; c—diabetic rats administered with ethanolic extract of *C. papaya*.

Figure 1a,b shows that the action of glucose-6-phosphatase and fructose-1,6 bisphosphatase was considerably high in Group 2 diabetic rats. Treatment with *C. papaya* in Group 3 exhibited high values close to normal, similar to that of the metformin-treated rats in Group 4. Group 5 dealt with *C. papaya* and in no way showed any significant differences. Figure 2a,b represents the activities of hexokinase and pyruvate kinase in control and experimental rats. When compared to Group 1 rats, skeletal muscle of diabetic rats had decreased pyruvate kinase and hexokinase activity. In Group 3 diabetic rats, treatment of *C. papaya* significantly elevated (*p* < 0.05) the levels of HK and PK, as well as in metformin-treated rats in Group 4.

### 3.2. Effect of C. papaya on mRNA Expression of IRS-1 and Akt

**Figure 3:**
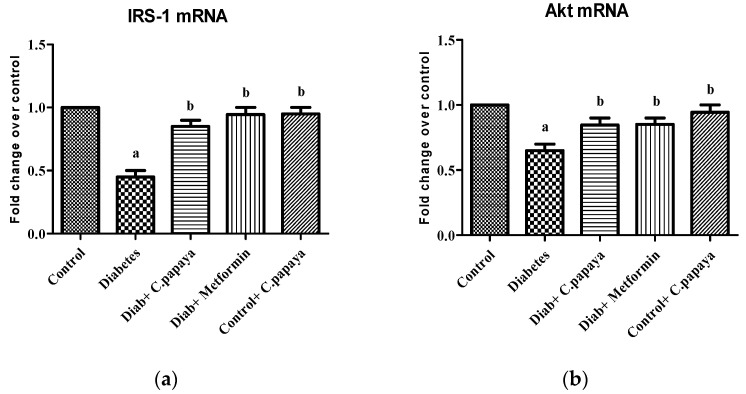
(**a**) IRS-1 mRNA expression levels in ethanolic extract of *C. papaya* in control and diabetic rats; (**b**) Akt mRNA expression levels in ethanolic extract of *C. papaya* in control and diabetic rats. Each bar demonstrates the mean ± SEM of eight rats, with *p* < 0.05 showing significant differences between the groups: a—control; b—diabetes.

The effect of *C. papaya* on mRNA expression on IRS-1 in skeletal muscle of all the five groups in the experimental study is portrayed in Figure 3a. There was a substantial decrease in the gene expression levels of IRS-1 in diabetic group rats. However, the treatment with *C. papaya* boosted IRS1 gene expression in skeletal muscle at par with the treatment of metformin. In skeletal muscle, Akt increased glycogen synthesis by activating glycogen synthase. In this study, Group 2 skeletal muscle had greatly reduced Akt gene expression. Interestingly, *C. papaya* administration revoked the gene levels of Akt, as shown in Figure 3b, even in the metformin treatment. No major alterations in Akt levels were seen in Groups 1 and 5. These results infer the capability of *C. papaya* to escalate the signaling of insulin in skeletal muscle of diabetic animals.

### 3.3. Evaluation of Immunohistochemical Changes in Skeletal Muscle

**Figure 4:**
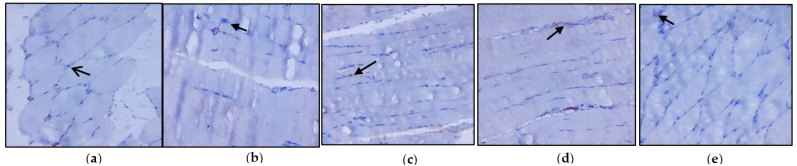
Protein expression of IRS-1 using an immunohistochemical assay (magnification: ×100): (**a**) control rats; (**b**) type 2 diabetic rats; (**c**) type 2 diabetic rats treated with *C. papaya* (600 mg/kg b.wt); (**d**) type 2 diabetic rats treated with metformin (50 mg/kg, b.wt); (**e**) control rats treated with *C. papaya* (600 mg/kg b.wt).

**Figure 5:**
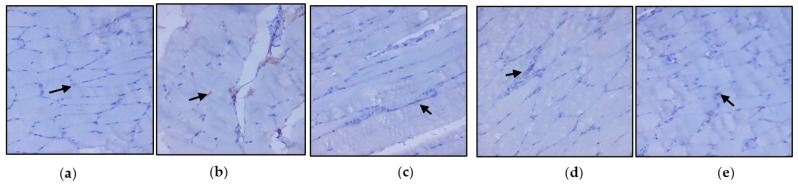
Protein expression of Akt using an immunohistochemical assay (magnification: ×100): (**a**) control rats; (**b**) type 2 diabetic rats; (**c**) type 2 diabetic rats treated with *C. papaya* (600 mg/kg b.wt); (**d**) type 2 diabetic rats treated with metformin (50 mg/kg, b.wt); (**e**) control rats treated with *C. papaya* (600 mg/kg b.wt).

Figure 4a–e represents the immunohistochemical changes in experimental and control rats. Group 2 animals showed decreased expression of IRS-1 in skeletal muscle. These levels were enhanced by the treatment of *C. papaya*, as seen in Group 3. Group 4 rats also exhibited a significant increase in IRS-1 expression in metformin treatment. Group 5 rats did not show any considerable changes. Figure 5a–e shows the Akt protein expression in the five groups studied. The diabetic group displayed lowered expression in skeletal muscle, and treatment with *C. papaya* improved the expression of Akt in a similar manner. Similarly, treatment with metformin showed a significant increase in Akt expression. The control rats treated with *C. papaya* did not show any significant changes in protein expression.

### 3.4. Molecular Docking

**Figure 6:**
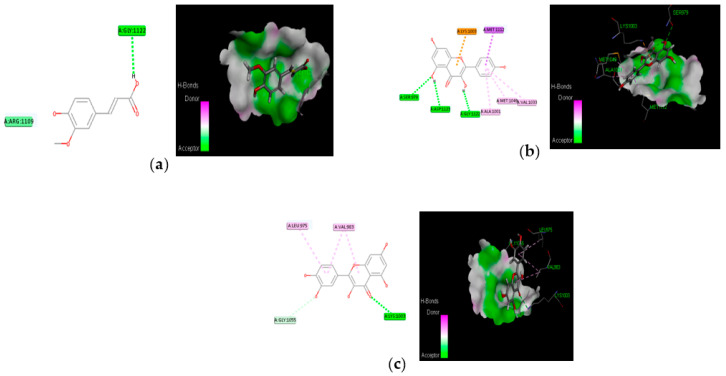
Molecular interaction of best three compounds with IRS-1; (**a**) trans-ferulic acid; (**b**) kaempferol; (**c**) quercetin.

**Figure 7:**
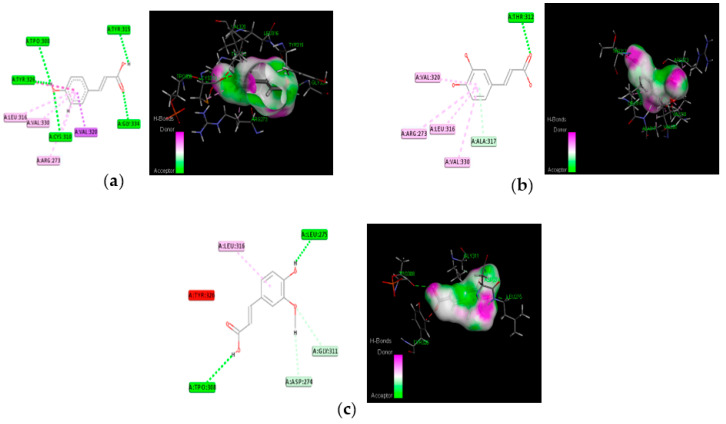
Molecular interaction of best three compounds with Akt; (**a**) p-coumaric acid; (**b**) caffeic acid; (**c**) trans-ferulic acid.

The proposed compounds were screened with the help of Discovery Studio software for their binding potentials on targets such as IRS-1 and Akt. The 3D structures of the targets were obtained from the RCSB Protein Data Bank. The results of this docking investigation revealed that the selected compounds have significant interaction with the target proteins. The protein–ligand complex’s stability was assessed using two key criteria: (1) the greatest binding energy and (2) the number of ligand interactions with the active site residues. Table 3 lists the docking details of all ligands with the target proteins. While docking into the active site, a ligand can experience van der Waals, hydrogen bonding, hydrophobic, and electrostatic interactions. According to the literature, binding energy plays a larger influence in predicting the optimum binding mode than the number of contacts. The traditional H bond (HB) (which is more prevalent) and hydrophobic contacts are more effective than the others. In our previous study, the results of ADME prediction were carried out in accordance with Lipinski’s rule of five. Rutin and chlorogenic acid failed to satisfy Lipinski’s rule of five, so we cannot consider these two compounds for further analysis. Table 3 shows the distinct binding pocket amino acid residues that are associated with the phytocompounds. The binding energy and number of interactions clearly show that selected compounds have a higher affinity for target proteins. The best three compounds for each target protein were chosen based on binding energy and interaction. Figure 6 and Figure 7 show the interaction of the best compounds with each target protein.

### 3.5. Molecular Simulation and Dynamics Study of Docked Complex

#### 3.5.1. Molecular Dynamic Simulation of IRS-1

**Figure 8:**
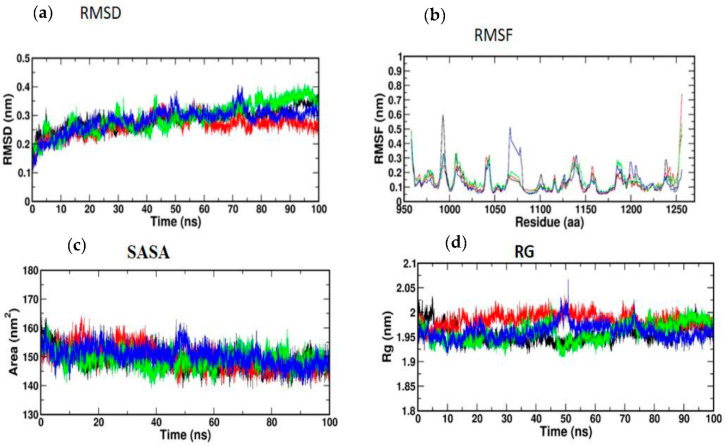
(**a**) RMSD; (**b**) RMSF; (**c**) SASA; (**d**) RG of IRS-1 protein with the top three compounds. Kaempferol shown in red; quercetin (green); trans-ferulic acid (blue).

Atomic RMSDs of the C atoms in an IRS-1–ligand complex, including kaempferol (red) and quercetin (green), as well as trans-ferulic acid (blue), were plotted in a time-based pattern, in addition to apo form (black) of IRS-1 protein, in a time-dependent manner (Figure 8a). Figure 8a showcases the stable nature of the apo form of IRS-1 after IRS-1 complexes with all compounds. Compared to other complexes, kaempferol only showed mild RMSD at 85 ns, after which it attained a stable form. Figure 8b shows the RMSF graph of IRS-1 with selected compounds. This plot shows that all the compounds showed stable form except trans-ferulic acid. Trans-ferulic acid showed a huge variation at the 1075 residues position, after which it attained a stable conformation. The evaluation of RG value for apoproteins and compounds (Figure 8d) was completed in order to investigate the binding impact of ligand to protein on compactness. RG values in the lower range indicate a strong association between the ligand and the protein, but the RG value of a stably folded protein is consistent. This plot demonstrated that the protein displayed a slight change of about 48–50 ns, after which it reached a stable state. In protein folding studies, the SASA of a protein is investigated as a critical element in the stability of protein, and it is found to be important. In this study, we determined the SASA values for the apo form of IRS-1 and for the proteins associated with each of the compounds, and the results are presented in Figure 8c.

#### 3.5.2. Molecular Dynamic Simulation of Akt

**Figure 9:**
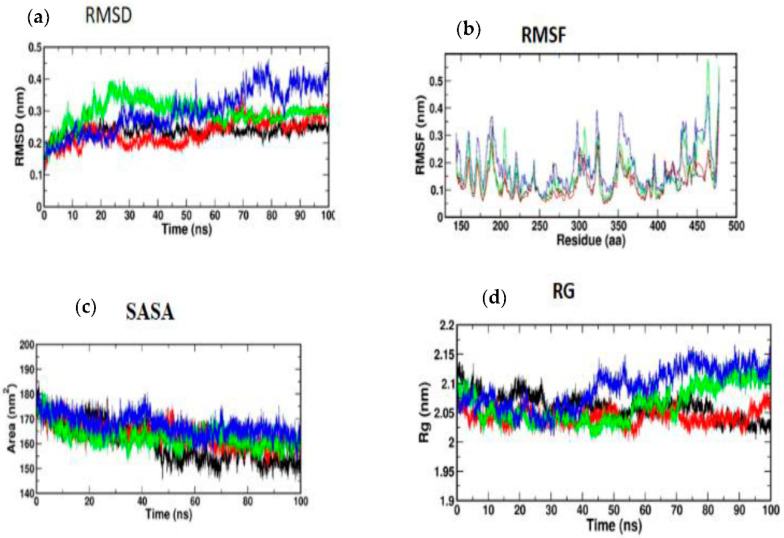
(**a**) RMSD; (**b**) RMSF; (**c**) SASA; (**d**) RG of Akt protein with the top three compounds. Caffeic acid shown in red; p-coumaric acid (green); trans-ferulic acid (blue).

According to Figure 9a, the MD simulation was carried out for a total time of 100 ns, and the trajectories are for the relative mean square deviation (RMSD) plot. apoprotein (black), caffeic acid (red), p-coumaric acid (green), and trans-ferulic acid (blue) are depicted in the illustration for a time scale of 100 ns. The RMSD of apoprotein showed a stable form throughout the dynamic simulation time. This plot showed that caffeic acid and p-coumaric acid fluctuated in a milder way, but trans-ferulic acid fluctuated at 75–85 ns; after that, it attained a stable conformation. Figure 9b shows the RMSF graph of docked complexes. Caffeic acid and p-coumaric acid did not show any abnormal fluctuation throughout the simulation time, but trans-ferulic acid showed a fluctuation at the 350–370 and 450 positions of amino acid residues. An additional measure of protein stability and compactness during MD tests can be found in the atoms’ RMSD from the protein centroid, which is called the radius of gyration (RG). Figure 9d shows the frequency of distribution of the values computed in each timestep for each simulation of the complexes. This plot showed that p-coumaric acid and trans-ferulic acid fluctuated at 48–100 ns of the simulation. The SASA profile of the compounds and receptor complex was equal throughout the simulation, indicating that the proteins have good interaction with the target protein (Figure 9c).

## 4. Discussion

The consumption of a high-fat diet leads to the development of diabetes mellitus, which progressively affects metabolic organs such as the liver, skeletal muscle, and adipose tissue. This results in an increase in glucose, cholesterol, triglycerides, and LDH and a decrease in HDL, causing hyperglycemia. In T2DM, hyperinsulinemia is closely associated with dysregulated insulin secretion and chronically elevated insulin levels in the bloodstream [41]. The induction of streptozotocin affects beta cell function and results in an abnormal insulin structure that eventually fails to bind with insulin receptors in the cells of target organs. This can pave the way to decrease the binding of the insulin receptor to insulin receptor substrates and thereby decrease downstream signaling pathways such as the PI3K, Akt, and AS 160. The translocation of GLUT4 transporters is reduced, eventually triggering lowered glucose uptake, causing reduced glucose metabolism [42]. In the present study, ethanolic extract of *C. papaya* administered to experimental rats improved insulin sensitivity in skeletal muscle of high-fat-diet–streptozotocin-induced T2DM.

Under normal conditions, insulin regulates glycolysis and gluconeogenesis, which initiates glucose uptake and oxidation in peripheral organs such as muscle and adipose tissues for the maintenance of normal blood glucose levels [43]. In diabetic conditions, the activity of enzymes related to these pathways fluctuates often, along with changes in glucose oxidation in metabolic organs that in turn induce insulin resistance in these organs [44]. Glucose-6-phosphatase and fructose-1,6 bisphosphatase are the key enzymes of gluconeogenesis. Activation of these enzymes is due to the state of insulin deficiency because, under normal conditions, insulin functions as a suppressor of gluconeogenic enzymes. The increase in the activity of gluconeogenic enzymes in T2DM produces hydrogen and combines with NADP+ to make NADPH and promote lipogenesis, which leads to higher blood glucose levels [45]. In our study, the levels of glucose-6-phosphatase and fructose-1,6 bisphosphonates were seen to be significantly elevated in the gastrocnemius muscles of diabetic animals. Administration of *C. papaya* to the diabetic rats decreased these enzymatic levels close to that of the metformin group. Similarly, in a study by Kanadi et al. [46], the seeds of *C. papaya* were found to eliminate potassium-bromate-induced alterations in the levels of glucose-6-phosphatase and fructose-1,6 bisphosphatase in rodent kidneys. A study evaluating the hypoglycemic property of *Centella asiatica* was conducted by Oyenihi and coworkers [47] to investigate the carbohydrate enzyme fructose-1,6 bisphosphate in skeletal muscle of rats and displayed that the activity of fructose-1,6 bisphosphate was increased by 23% with the administration of *Centella asiatica* in the diabetes-induced rats. Additionally, isopulegol was also studied for its hypoglycemic property in the liver of diabetes-induced rodents by Kalaivani et al. [48]. The present study demonstrated for the first time the action of *C. papaya* on gluconeogenic enzymes viz. glucose-6-phosphatase and fructose-1,6 bisphosphatase in skeletal muscle of high-fat-diet–streptozotocin-induced diabetic rats.

Insulin secretion and the metabolic processes of different cells are normally influenced by glycolysis. HK and PK are key enzymes of glycolysis, and their deficiency can result in reduced glycolysis and reduced uptake and utilization of glucose for energy production, which can lead to insulin resistance [45,49]. In our present study, HK and PK were decreased in high-fat-diet–streptozotocin-induced diabetic rats due to faulty insulin signaling. The administration of *C. papaya* elevated the levels of these glycolytic enzymes in skeletal muscle of diabetic rats when compared to the metformin group. Pari et al. [50] mentioned that the phytocompound coumarin also had a similar property that increased the levels of glycolytic enzymes in diabetic rats as compared to that of the control group. Likewise, Gothandam et al. [51] showed elevated levels of glycolytic enzymes in diabetic skeletal muscle by theaflavins. Our study showed that the antidiabetic properties of *C. papaya* may be due to its phytochemical potential to reduce blood glucose and regulate insulin signaling [52].

Insulin signaling plays a vital role in the control of a wide range of biological processes such as glucolipid homeostasis, predominantly via action on metabolic organs [53]. Tyrosine autophosphorylation of the β-subunit, which occurs as a result of insulin binding to the insulin receptor, phosphorylates other substrates and triggers a signaling cascade to oxidize glucose for energy production. Disturbances in these signaling pathways can lead to insulin resistance [54]. A high-fat diet disrupts insulin signaling by affecting glucose and lipid homeostasis, which changes the regular functioning of insulin signaling molecules and results in insulin resistance [55]. Specifically, in skeletal muscle, where 80% of glucose oxidation takes place, insulin signaling molecule alteration may reflect more when compared to other organs such as the liver and adipose tissue [56].

IRS-1, which is specific in peripheral tissues such as skeletal muscle and adipose tissue, plays a significant role in insulin signaling pathways. In diabetic conditions, the insulin binds to the insulin receptor inefficiently, leading to serine phosphorylation of IRS-1 instead of tyrosine phosphorylation, which lowers IRS-1 activation, leading to decreased activation of downstream insulin signaling molecules such as PI3 kinase, Akt, and AS160 [57]. Our study showed for the first time the action of *C. papaya* on insulin signaling molecules such as IRS-1 in skeletal muscle of high-fat-diet–streptozotocin-induced diabetic rats. This study demonstrated that the mRNA levels of IRS-1 in the diabetic group are lowered when compared to the control group. Diminished IRS-1 activity may be due to the altered action of gluconeogenic and glycolytic enzymes, which in turn caused less binding of the insulin receptor to IRS-1. This subsequently reduced the activation of Akt. Zhang et al. [58] showed upregulation of mRNA levels of IRS-1 by fucoxanthin administration in diabetic skeletal muscle. Another study conducted by Cai et al. [59] showed enhanced gene expression levels of IRS-1 by *Folium Mori* in T2DM skeletal muscle. The treatment with *C. papaya* showed an increase in the levels of IRS-1 compared to metformin and displayed the mechanism in its antidiabetic nature.

A high-fat diet-induced state of insulin resistance also regulates the activation of Akt, a serine-threonine kinase that controls cellular signaling pathways. This current study showed the action of *C. papaya* on Akt in skeletal muscle of the high-fat-diet–streptozotocin-induced diabetic rats. This study demonstrated that the mRNA levels of Akt in the diabetic group are lowered when compared to the control group, whereas the *C. papaya*-treated group showed a significant change in mRNA levels, indicating *C. papaya* contains phytochemicals that enhance insulin signaling molecules such as Akt for further normal glucose uptake and oxidation. The phytocompound saponins in *Panax notoginseng* regulates the mRNA levels of Akt in skeletal muscle of T2DM mice according to Guo et al. [60]. Likewise, Jung et al. [61] reported that the treatment with asprosin improved insulin sensitivity by upregulating levels of insulin receptor substrate 1 and Akt phosphorylation. The high-fat-diet condition influenced these insulin signaling molecules, which further affected the translocation of GLUT4 through AS160, causing a decrease in glucose uptake that results in decreased glucose oxidation in peripheral tissues [62]. The above reports in our study hint at the possible signaling mechanism by which *C. papaya* exerts its antidiabetic property.

The antidiabetic property of *C. papaya* and its enhancement on the IRS-1 and Akt are strengthened by immunohistochemical studies. In our study, the treatment with *C. papaya* was effective in boosting up these protein targets involved in the insulin signaling pathway comparable to that of metformin and thereby increased insulin sensitivity in skeletal muscle. In diabetic skeletal muscle, the effect of staining of these molecules was very much reduced when compared to control rats. Wang et al. [63] reported increased IRS-1 degradation in the fatty tissue of a type 2 diabetic rodent model, and they suggested a faulty glucose uptake due to defective GLUT4. Li et al. [64] demonstrated that treatment with dioscin regulated the levels of IRS-1 and Akt close to the normal group thIt triggered the insulin signaling. The effect of ethanolic extract of *C. papaya* in regulating the gluconeogenic and glycolytic enzymes, as well as in the enhancement of mRNA levels of IRS-1 and Akt, could possibly be explained in the immunohistochemical staining in our study. 

The discovery of active molecules from natural sources has risen to prominence as a critical component of drug discovery. The docking and dynamic studies in our work were conducted to count on the compound’s antidiabetic efficacy at the target level. However, for binding affinity analysis, the scores were transferred from the table browser view of Discovery Studio for the top-ranked docked complex. We identified and proposed antidiabetic peptides for oral administration, employing an in silico method in our work. Trans-ferulic acid had the greatest hit rate against the protein targets IRS-1 and Akt, which may act as adjuvant drugs to tackle T2DM with lesser or no complications and need to be validated by wet-lab investigations.

## 5. Conclusions

*C. papaya* has the capability to normalize blood glucose levels in diabetic rats, and it was shown to reinstate the insulinemic effect in diabetic skeletal muscle by boosting IRS-1 and Akt levels. The novelty of this study is that we are the first to describe the conceivable role of *C. papaya* on insulin signaling molecules such as IRS-1 and Akt in a high-fat-diet–streptozotocin T2DM model. Furthermore, we also proposed via molecular docking and dynamics that trans-ferulic acid of *C. papaya* docked well with IRS-1 and Akt. The results of in silico studies also supported our experimental studies. Therefore, considering the above findings, it is evident that *C. papaya* may be a reassuring drug for T2DM management.

## Figures and Tables

**Table 1:** Primer sequence.

S. No	Gene Name	Primer Sequence	Reference
1	Rat βactin	Sense primer: 5′-AAG TCC CTC ACC CTC CCA AAA G-3′ Antisense primer: 5′-AAG CAA TGC TGT CAC CTT CCC-3′	[35]
2	IRS-1	Sense primer: 5′-GCC AAT CTT CAT CCA GTT GCT-3′ Antisense primer: 5′-CAT CGT GAA GAA GGC ATA GGG-3	[36]
3	Akt	Sense primer: 5′-GGA AGC CTT CAG TTT GGA TCC CAA-3′ Antisense primer: 5′-AGT GGA AAT CCA GTT CCG AGC TTG-3′	[37]

**Table 2:** List of selected compounds from *C. papaya*.

S. No.	Compound Name
1	Caffeic_acid
2	Chlorogenic_acid
3	Kaempferol
4	Quercetin
5	Rutin
6	p-coumaric_acid
7	trans-ferulic_acid

**Table 3:** Binding affinity assessment based on docking score of proposed natural compounds and selected target proteins.

S. No	Compound Name	Lig Score1_Drei Ding	Lig Score2_Drei Ding	PLP 1	PLP 2	JAIN	PMF	Docking Score
IK3A								
1	Trans-ferulic acid	1.64	3.37	38.93	36.6	−1.2	34.9	37.161
2	Quercetin	2.69	3.56	52.33	58.2	−0.84	52.63	49.741
3	Kaempferol	0.32	1.75	52.03	65.41	0.75	67.22	49.413
4	Rutin	3.33	4.24	109.67	113.31	1.14	73.52	103.327
5	p-coumaric acid	No interaction						
6	Chlorogenic acid	3.96	4.6	75.2	75.63	−0.37	64.06	71.235
7	Protocatechuic acid	No interaction						
8	Caffeic acid	No interaction						
3QKM								
1	Trans-ferulic acid	1.02	0.14	64.19	71.24	2.36	−8.12	58.136
2	Quercetin	−18.41	−31.47	9.64	54.3	5.87	−37.75	0.656
3	Kaempferol	−16.08	−28.66	12.82	52.39	6.57	−27.04	4.939
4	Rutin	No interaction						
5	p-coumaric acid	0.26	−0.99	55.11	62.57	3.43	7.61	50.999
6	Chlorogenic acid	No interaction						
7	Protocatechuic acid	No interaction						
8	Caffeic acid	−2.19	−4.55	54.21	59.96	3.52	−2.24	51.777

## Data Availability

The data presented in this study are available in this article.
